# Plasma 4β-Hydroxycholesterol Predicts Ribociclib CYP3A-Mediated Metabolism, but Not Plasma Exposure, in Patients with Primary Brain Cancer

**DOI:** 10.3390/cancers18162667

**Published:** 2026-08-18

**Authors:** Charuka Wickramasinghe, Xun Bao, Yuanyuan Jiang, Jun Jiang, Yang Yue, Nader Sanai, Jing Li

**Affiliations:** 1 Karmanos Cancer Institute, Wayne State University School of Medicine, 4100 John R Street, HWCRC, Room 523, Detroit, MI 48201, USA; 2 Barrow Neurological Institute, St. Joseph’s Hospital & Medical Center, Phoenix, AZ 85004, USA

**Keywords:** ribociclib, LEQ803, 4β-hydroxycholesterol (4β-OHC), endogenous biomarker of hepatic CYP3A4, parent-metabolite population pharmacokinetic model

## Abstract

**Background/Objectives:** This study aimed to evaluate the potential utility of plasma 4β-hydroxycholesterol (4β-OHC) as an endogenous biomarker of hepatic CYP3A activity for predicting ribociclib metabolism and systemic pharmacokinetics in patients with primary brain cancer. **Methods:** Plasma concentration data for ribociclib and its major metabolite, LEQ803, were obtained from 46 patients receiving oral ribociclib at daily doses of 400, 600, or 900 mg for 5 days. Plasma 4β-OHC concentrations and 4β-OHC/cholesterol ratios were determined by LC–MS/MS at baseline and at 4 and 24 h after ribociclib administration on day 5. A parent–metabolite population pharmacokinetic model was developed to simultaneously characterize ribociclib and LEQ803 disposition and identify clinical covariates influencing their pharmacokinetics. **Results:** The parent–metabolite model adequately described the multiple-dose plasma concentration–time profiles of ribociclib and LEQ803. On-treatment plasma 4β-OHC concentration was identified as a significant predictor for CYP3A-mediated ribociclib metabolism to LEQ803, accounting for ~36% of interindividual variability in the metabolic clearance. Plasma 4β-OHC showed limited ability to predict total apparent oral clearance or systemic exposure of ribociclib. **Conclusions:** These findings support the clinical utility of plasma 4β-OHC as an endogenous biomarker of hepatic CYP3A activity for predicting CYP3A-mediated metabolism of substrate drugs such as ribociclib, while highlighting its limitation for predicting total apparent oral clearance of drugs undergoing intestinal first-pass metabolism and elimination through multiple pathways. These results underscore the importance of considering the relative contribution of hepatic CYP3A metabolism to overall drug disposition when evaluating translational utility of endogenous CYP3A biomarkers in clinical pharmacology.

## Simple Summary

Ribociclib, a cyclin-dependent kinase 4/6 (CDK4/6) inhibitor, exhibits favorable central nervous system (CNS) penetration, supporting its clinical development for brain cancer treatment. However, substantial interindividual variability in systemic exposure may affect CNS drug penetration and therapeutic response. To investigate determinants of this variability, a parent–metabolite population pharmacokinetic model was developed that adequately characterized the plasma concentration–time profiles of ribociclib and its major metabolite, LEQ803, and identified clinical covariates influencing their pharmacokinetics. On-treatment plasma 4β-hydroxycholesterol (4β-OHC), an endogenous biomarker of hepatic CYP3A activity, was identified as a significant predictor of CYP3A-mediated ribociclib metabolism to LEQ803. In contrast, plasma 4β-OHC showed limited ability to predict total apparent oral clearance or systemic exposure of ribociclib. These findings improved our understanding of the factors underlying ribociclib pharmacokinetic variability and provided clinically meaningful insights to support the rational development and optimal use of ribociclib in neuro-oncology.

## 1. Introduction

Cyclin-dependent kinase 4 and 6 (CDK4/6) inhibitors have transformed the treatment landscape for hormone receptor-positive breast cancer and being investigated in other malignancies characterized by dysregulated cell cycle signaling, including primary brain cancer [1,2,3,4,5,6,7]. Among these agents, ribociclib has demonstrated favorable central nervous system (CNS) penetration in glioblastoma patients in early clinical studies [1,3,5]. However, despite favorable CNS pharmacokinetics and good tolerability, ribociclib monotherapy has demonstrated limited clinical efficacy in patients with glioblastoma [5,8]. In addition to biological mechanisms (such as signaling pathway redundancy in glioblastoma) that may limits its efficacy, substantial interindividual variability in ribociclib systemic exposure may contribute to differences in CNS drug delivery and ultimate therapeutic response.

Ribociclib is predominantly metabolized by hepatic cytochrome P450 3A (CYP3A), leading to a major metabolite M4 (LEQ803, *N*-desmethyl ribociclib) and several other metabolites; in addition, it is metabolized, to a less extent, by flavin-containing monooxygenase 3 (FMO3) to metabolite M13 (CCI284, *N*-hydroxylation) [9]. Given the predominant role of CYP3A-mediated metabolism in ribociclib disposition, variability in CYP3A activity arising from genetic, physiological, pathological, or drug-related factors may substantially alter ribociclib pharmacokinetics. In patients with brain cancer, factors such as corticosteroid use, antiepileptic drugs, inflammation, hepatic dysfunction, and polypharmacy may further complicate CYP3A-mediated drug metabolism, thereby leading to a wider interindividual variability in ribociclib systemic exposure. Clinical studies have demonstrated that ritonavir (a strong CYP3A inhibitor) increased ribociclib area under the plasma concentration–time curve (AUC) of a single 400 mg dose by 3.2-fold, whereas rifampin (a strong CYP3A inducer) decreased ribociclib AUC by 89% in healthy volunteers [10]. Because CYP3A activity in patients with brain cancer can be substantially altered by polypharmacy and concomitant medications, considerable interindividual variability in ribociclib systemic exposure is expected. As systemic drug exposure drives drug CNS penetration, identification of an endogenous CYP3A activity biomarker that could predict systemic pharmacokinetics or exposure of ribociclib would therefore facilitate therapeutic dosing optimization for rational development of this CDK4/6 inhibitor in patients with brain cancer.

The exogenous probe substrate midazolam has been validated as the gold standard for phenotyping in vivo CYP3A activity [11,12,13]. However, their clinical application in oncology patients is limited by the need for exogenous drug administration, multiple blood sampling, and potential safety concern. In contrast, 4β-hydroxycholesterol (4β-OHC), an endogenous oxysterol formed primarily through CYP3A-mediated oxidation of cholesterol, has emerged as a promising minimally invasive biomarker of in vivo CYP3A activity [14,15,16]. Once formed, 4β-OHC exhibits remarkable metabolic stability, with a long plasma half-life ranging from approximately 60 h to 17 days [17]. This prolonged stability enables 4β-OHC to accumulate in plasma at relatively steady concentrations, making it well suited for longitudinal monitoring of CYP3A activity. Its stability also reduces intra-individual variability, thereby enhancing its reliability as a biomarker. Importantly, 4β-OHC measurement does not require administration of exogenous compounds and can be readily incorporated into clinical pharmacology studies using opportunistically collected plasma samples. Previous studies have demonstrated significant associations between plasma 4β-OHC concentrations (or 4β-OHC/cholesterol ratios) and the pharmacokinetics of CYP3A substrates such as midazolam [14,18,19], supporting its utility as a noninvasive phenotyping marker. However, because 4β-OHC is produced predominantly in the liver, it may not adequately reflect total CYP3A activity across all tissues, particularly intestinal CYP3A activity [20,21].

Given the critical role of CYP3A in ribociclib disposition, this study aimed to evaluate the potential utility of plasma 4β-OHC as an endogenous CYP3A biomarker for predicting ribociclib metabolism and systemic pharmacokinetics in patients with primary brain cancer. Specifically, a parent–metabolite population pharmacokinetic model was developed to characterize the plasma concentration–time profiles of ribociclib and its major metabolite, LEQ803, and identify clinical covariates influencing the plasma pharmacokinetics of ribociclib and LEQ803 in patients. Although ribociclib has promising therapeutic potential for the treatment of brain malignancies, including glioblastoma and breast cancer brain metastases, its clinical efficacy in these settings has yet to be fully established. A better understanding of the determinants of the pharmacokinetic variability of ribociclib is essential for optimizing systemic drug exposure, maximizing CNS drug delivery, and ultimately improving therapeutic outcomes in patients with brain cancer. By identifying an endogenous biomarker predictive of CYP3A-mediated ribociclib metabolism, our study provided clinically meaningful insights to support the rational development and optimal use of ribociclib in neuro-oncology.

## 2. Materials and Methods

### 2.1. Clinical Study and Pharmacokinetic Data

The plasma concentration data of ribociclib and LEQ803 were obtained from two Phase 0/II studies (ClinicalTrials.gov ID: NCT02933736 and NCT03834740), which were conducted at the Ivy Brain Tumor Center, Barrow Neurological Institute, St. Joseph’s Hospital and Medical Center, Phoenix, AZ. The protocols were approved by the Institutional Review Board of St. Joseph’s Hospital and Medical Center. Written informed consents were obtained from all participants. In NCT02933736 study, 20 patients (12 with glioblastoma and 8 with meningioma) were enrolled and treated with oral ribociclib 900 mg once daily for 5 days preceding planned cranial tumor resection [5]. In NCT03834740 study, 26 patients with glioblastoma were enrolled and treated with ribociclib 400 or 600 mg once daily for 5 days plus everolimus (at the dose ranging from 2.5 to 10 mg daily for 5 days or a single higher dose on day 5 at 50–70 mg) preceding planned cranial tumor resection [1].

Eligible patients were required to have newly diagnosed or recurrent high-grade glioma or meningioma and meet the following criteria: age > 18 years, Eastern Cooperative Oncology Group (ECOG) performance status ≤ 2, and adequate bone marrow and organ functions. Adequate bone marrow function was defined as absolute neutrophil count ≥ 1.5 × 10^9^/L, platelet count ≥ 100 × 10^9^/L, and hemoglobin ≥ 9 g/dL. Adequate liver function was defined as serum transaminase (AST and ALT) levels ≤ 3 times the institutional upper limit of normal (ULN) and total bilirubin ≤ 1.5 × ULN. Adequate kidney function was defined as the glomerular filtration rate (eGFR) ≥ 30 mL/min/1.73 m^2^ estimated by Chronic Disease Epidemiology Collaboration (CKD-EPI) equation, and either serum creatinine ≤ 1.5 × ULN or creatinine clearance ≥ 60 mL/min for those with serum creatinine levels above 1.5 × ULN. Demographic and characteristic data for all patients are summarized in Table 1.

In both studies, blood samples were collected at pre-dose and at 0.5, 1, 2, 4, 7, and 24 h following the administration of ribociclib on day 5. Plasma was separated from whole blood by centrifugation at 1000 *g* and 4 °C for 10 min and stored at −80 °C until analysis. The plasma concentrations of both ribociclib and LEQ803 (*N*-desmethyl ribociclib) were quantified in 26 patients receiving ribociclib 400 or 600 mg daily (NCT03834740 study), while plasma concentrations of ribociclib only were quantified in 20 patients receiving ribociclib 900 mg daily (NCT02933736 study), using a validated liquid chromatography coupled with tandem mass spectrometry (LC-MS/MS) method [22].

### 2.2. LC-MS/MS Quantitation of Plasma 4β-Hydroxycholesterol and Cholesterol Concentrations

Plasma concentrations of 4β-hydroxycholesterol (4β-OHC) and cholesterol were determined in patient plasma samples collected at the baseline (pre-treatment) and at 4 and 24 h following ribociclib administration on day 5, using a validated LC-MS/MS method. Briefly, to determine cholesterol, plasma samples were diluted 100 folds by water and subjected to protein precipitation with 5 volumes of methanol containing the internal standard, D7 cholesterol (Cayman Chemicals, Ann Arbor, MI, USA). The resulting supernatant was analyzed by LC-MS/MS. To determine 4β-OHC, plasma samples were precipitated with 5 volumes of methanol containing the internal standard, D7 4β-OHC (Cayman Chemicals, Ann Arbor, MI, USA). The resulting supernatant was subsequently dried-down in a CentriVap^®^ Refrigerated Centrifugal Concentrator (Kansas City, MO, USA) at 10 °C. The residue was reconstituted in methanol followed by vortex-mixing and centrifugation, and the supernatant was analyzed by LC-MS/MS.

LC-MS/MS analysis was performed on an AB SCIEX QTRAP 6500 LC-MS/MS system. Chromatographic separation was achieved on a KINETEX Core–shell C18 column (Phenomenex, Torrance, CA, USA, 150 × 2.1 mm, 2.6 µm, 100 Å) under a gradient elution consisting of mobile phase A (0.1% formic acid in water) and mobile phase B (0.1% formic acid in methanol), at a flow rate of 0.4 mL/min. Cholesterol, 4β-OHC, and their respective internal standards were monitored under the positive atmospheric pressure chemical ionization (APCI) mode at the mass transitions of 369.4 > 147.1, 385 > 367.5, 376.4 > 147.4, and 392 > 374.5, respectively. Calibration curves were prepared in water over concentration ranges of 0.002 to 2 µM for 4β-OHC and 0.1 to 100 µM for cholesterol. The intra- and inter-day variations in all quality control samples were within 15%.

### 2.3. Population Pharmacokinetic Model Development and Evaluation

A parent-metabolite population PK model was developed to simultaneously characterize the plasma concentration–time profiles of ribociclib and LEQ803 following 5 days of daily oral doses at 900, 600, or 400 mg. The population PK model was developed in two steps: (1) basic (structural) model development and (2) covariate model development. All analyses were performed using the nonlinear mixed-effects modeling program MONOLIX version 2024R1 (Lixoft, Antony, France).

**Figure 1:**
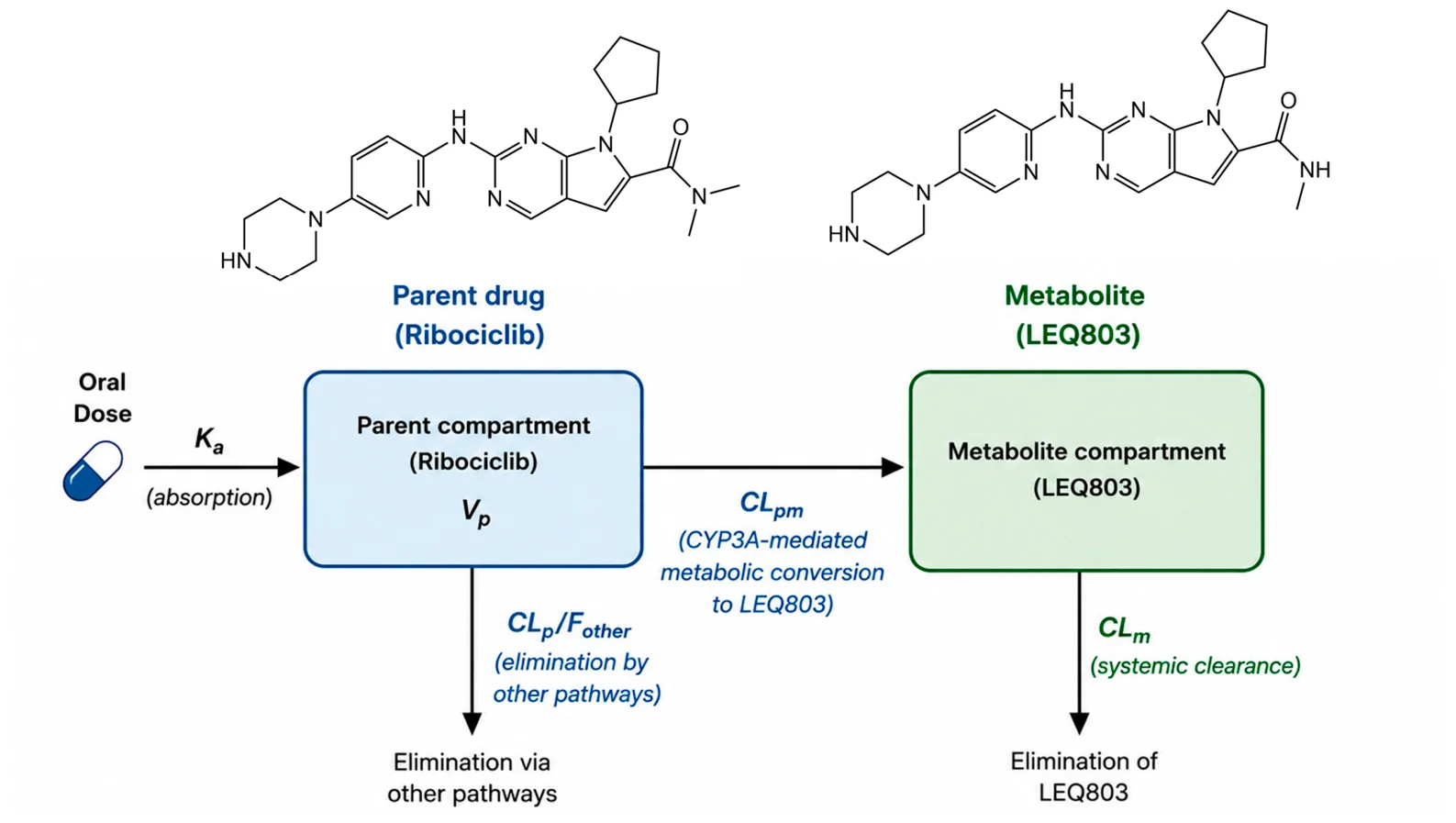
Model structure of the parent-metabolite pharmacokinetic model.

***Structure Model Development*****.** The parent–metabolite model (Figure 1) comprised a parent drug compartment (Vp) and a metabolite compartment (Vm). Following oral administration, ribociclib was absorbed into the parent compartment at a first-order absorption rate constant (Ka), and was eliminated through CYP3A-mediated metabolic conversion to LEQ803, characterized by the metabolic clearance (CLpm), and elimination via all other pathways, represented by the apparent clearance (CLp/F_other_). LEQ803 was formed from ribociclib and subsequently eliminated from the metabolite compartment by its systemic clearance (CLm). The model was developed to simultaneously fit log-transformed plasma concentration data of ribociclib and LEQ803 from all 46 patients. Population mean pharmacokinetic parameters, interindividual variability, and residual error were assessed in the model. Interindividual variability was modeled using an exponential function, assuming a log-normal distribution for all parameters. Various residual error models, including constant, proportional, and combined error models, were evaluated. The combined error model, incorporating both proportional and additive error components, was ultimately selected as the optimal residual error structure based on model performance. Population mean pharmacokinetic parameters were estimated using the Stochastic Approximation Expectation–Maximization (SAEM) algorithm, a robust approach for nonlinear mixed-effects modeling. Individual patient pharmacokinetic parameters were estimated using post hoc Bayesian estimation based on conditional distributions generated through the Markov Chain Monte Carlo (MCMC) procedure.

**Figure 2:**
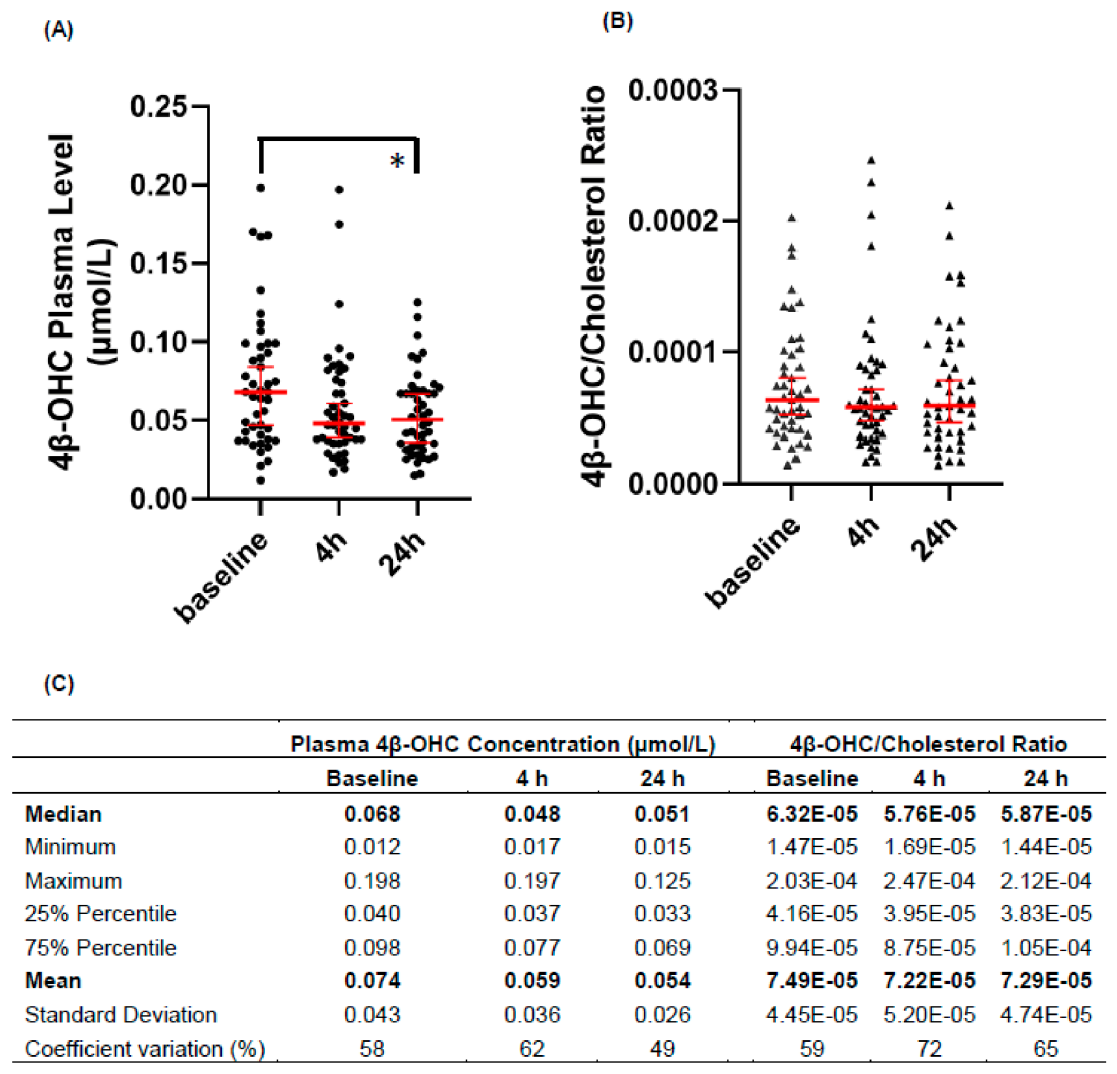
Endogenous biomarkers of hepatic CYP3A activity. (**A**,**B**) Plasma 4β-OHC concentrations and 4β-OHC/cholesterol ratios measured at baseline (pretreatment, day 0) and at 4 and 24 h following ribociclib administration on day 5 in 46 patients from two clinical trials. (**C**) Descriptive statistics of the measurements. * The reduction in plasma 4β-OHC concentrations at 24 h postdosing reached statistical significance (ANOVA with Tukey’s multiple-comparison test, adjusted *p* = 0.0208).

***Covariate Model Development.*** A screen for potential covariates was performed using R programming (version 4) with generalized additive model (GAM), a stepwise multiple regression method allowing covariates to enter into the model as either linear or non-linear fashion according to a natural cubic spline function with one internal break-point [23]. The covariates including sex, age, body size (i.e., weight, height, and BSA), plasma albumin levels, liver function (e.g., ALT, AST, and total bilirubin), and kidney function (e.g., serum creatinine and creatinine clearance) (Table 1), as well as in vivo hepatic CYP3A activity markers (e.g., plasma 4β-OHC level and 4β-OHC/cholesterol ratio) (Figure 2), were screened on post hoc Bayesian-estimated pharmacokinetic parameters from the structural model. For patients with missing covariate data, values were imputed using the population median, as the proportion of missing data was low (<2%). Continuous covariates were centered around their median values, while categorical covariates were encoded as binary variables (e.g., male = 1 and female = 0).

Potentially covariates selected from GAM analysis were introduced into the covariate model as linear, exponential, or power function, using a stepwise covariate modeling procedure. Covariate effects were found to be best described by exponential function (Equation (1)) or power function (Equation (2)).

(1) $$\theta_{i}=\theta_{pop}\times e^{\beta_{COV}\times {COV}_{j}}$$

(2) $$\theta_{i}=\theta_{pop}\times {COV}_{j}^{\beta_{COV}}$$

where $\theta_{i}$ represents a particular pharmacokinetic parameter for the $i^{th}$ patient, $\theta_{pop}$ is the population mean population value, $\beta_{COV}$ is the covariate effect coefficient, and ${COV}_{j}$ is the covariate.

In the step-wise covariate model building process, discrimination among hierarchical models was based on both statistical and graphical criteria. Specifically, model comparisons were first performed using the likelihood ratio test, in which reductions in the objective function value (OFV, −2 log-likelihood [−2LL]) were used to evaluate improvements in model fit. A covariate was retained if the addition of the covariate decreased OFV by >3.875 (*p* < 0.05) during the forward covariate model building, and the removal of the covariate increased the OFV by >10.828 (*p* < 0.001) during the stepwise backward model reduction [24]. Additional criteria for covariate inclusion included: (1) reduction in relative standard errors of parameter estimates, (2) decrease in interindividual variability, and (3) random scatter of points around a horizontal line of identity at 0 in plots of weighted residue versus predicted concentrations.

***Model evaluation*****.** The precision and stability of parameter estimates were evaluated using a nonparametric bootstrap approach implemented in MONOLIX (2024R1). In this procedure, multiple resampled datasets were generated by random sampling with replacement from the original dataset. Model parameters were re-estimated for each resampled dataset, and the process was repeated 500 times to generate empirical distributions of the parameter estimates [25,26]. The median values and corresponding 95% confidence intervals (CIs) of the bootstrap estimates were calculated to evaluate parameter robustness [26]. Agreement between the original parameter estimates and the mean values obtained from the bootstrap replicates indicate that the model is stable and parameter uncertainty is adequately captured [25,26,27].

The predictive performance of the developed parent–metabolite model was assessed using visual predictive checks (VPCs), in which 500 datasets were simulated based on the estimated population parameters and compared with the observed concentration–time data [28,29]. The simulated 50th percentile (median) and the 5th and 95th percentiles (corresponding to the 95% prediction interval) were overlaid with the observed concentrations. Adequate model performance was indicated when the majority of observed data points fell within the simulated 95% prediction interval and the observed percentiles aligned closely with the simulated percentiles. Conversely, systematic deviations between observed and simulated percentiles were considered indicative of potential model misspecification or the need for further model refinement.

In addition, prediction distribution plots were generated to compare the observed concentration data with the theoretical distribution of model-predicted concentrations [30]. Unlike VPCs, these plots are based on multiple simulations of the study individuals without residual error, focusing on the variability arising from structural and random effects components alone [30]. The simulations incorporated the estimated fixed and random effects from the population model while excluding parameter uncertainty; and therefore, prediction distribution plots enabled identification of potential systematic discrepancies between observed and predicted concentrations, particularly those arising from model misspecification, overdispersion, or unexplained variability [30].

### 2.4. Statistical Analysis

In vivo hepatic CYP3A activity, assessed by plasma 4β-OHC concentrations or 4β-OHC/cholesterol ratios, was compared among the baseline, 4 and 24 h after ribociclib administration on day 5 using one-way analysis of variance (ANOVA) followed by Tukey’s multiple-comparison post hoc test. Bivariate relationships between in vivo hepatic CYP3A activity biomarkers and pharmacokinetic parameters were evaluated using Spearman’s rank correlation analysis. Statistical significance was defined as a two-sided *p* value < 0.05. All statistical analyses were performed using GraphPad Prism (version 11).

## 3. Results

### 3.1. Endogenous Biomarker of Hepatic CYP3A Activity

Plasma 4β-OHC concentrations and 4β-OHC/cholesterol ratios measured at baseline (pretreatment, day 0) and at 4 and 24 h following ribociclib administration on day 5 in 46 patients from two clinical trials are summarized in Figure 2. In vivo hepatic CYP3A activity, as assessed by plasma 4β-OHC concentrations or 4β-OHC/cholesterol ratios, exhibited up to 16-fold interindividual variability across patients. Such substantial variability in hepatic CYP3A activity is likely to contribute to the pronounced pharmacokinetic variability observed for CYP3A substrate drugs. Compared with baseline, in vivo hepatic CYP3A activity appeared to decrease after 5 days of daily ribociclib treatment; particularly, the reduction in plasma 4β-OHC concentrations at 24 h postdosing reached statistical significance (ANOVA with Tukey’s multiple-comparison test, adjusted *p* = 0.0208). This finding is consistent with previous reports demonstrating that ribociclib acts as a time-dependent inhibitor of CYP3A [10].

### 3.2. Population Pharmacokinetic Model

Plasma pharmacokinetics of ribociclib were evaluated in 20 patients receiving ribociclib alone and in 26 patients receiving the combination of ribociclib and everolimus. No pharmacokinetic interactions were identified between ribociclib and everolimus. The multiple-dose plasma pharmacokinetic profiles of ribociclib and LEQ803 following daily oral administration of ribociclib at the dose of 400, 600, and 900 mg were adequately characterized by a parent-metabolite population pharmacokinetic model (Figure 1). The 24 h plasma 4β-OHC concentration was identified as the significant covariate on the metabolic clearance (CLpm) governing conversion of ribociclib to LEQ803, and patient height were identified as the significant covariates on the apparent clearance thought all other pathways (CLp/F_other_).

**Figure 3:**
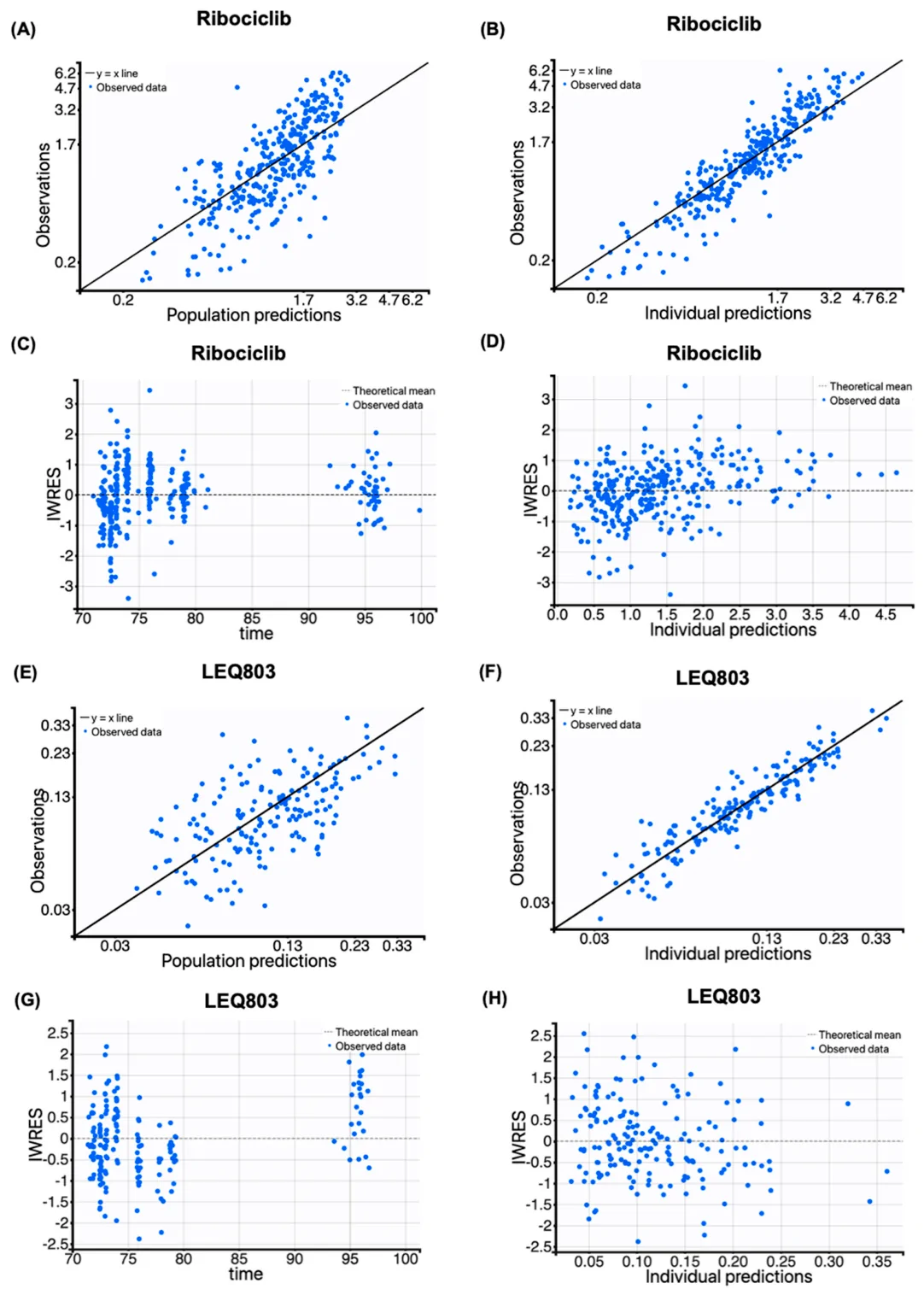
Goodness-of-fit plots for ribociclib and LEQ803 from the final covariate model. (**A**,**B**) The relationship between the observed and population parameter- or individual parameter-predicted ribociclib concentrations. The solid line is the line of identity. (**C**,**D**) Individual weighted residues (IWRESs) versus time or versus individual parameter-predicted ribociclib concentrations. The dashed line is the mean IWRES. (**E**,**F**) The relationship between the observed and population parameter- or individual parameter-predicted LEQ803 concentrations. The solid line is the line of identity. (**G**,**H**) Individual weighted residues (IWRESs) versus time or versus individual parameter-predicted LEQ803 concentrations. The dashed line is the mean IWRES.

**Figure 4:**
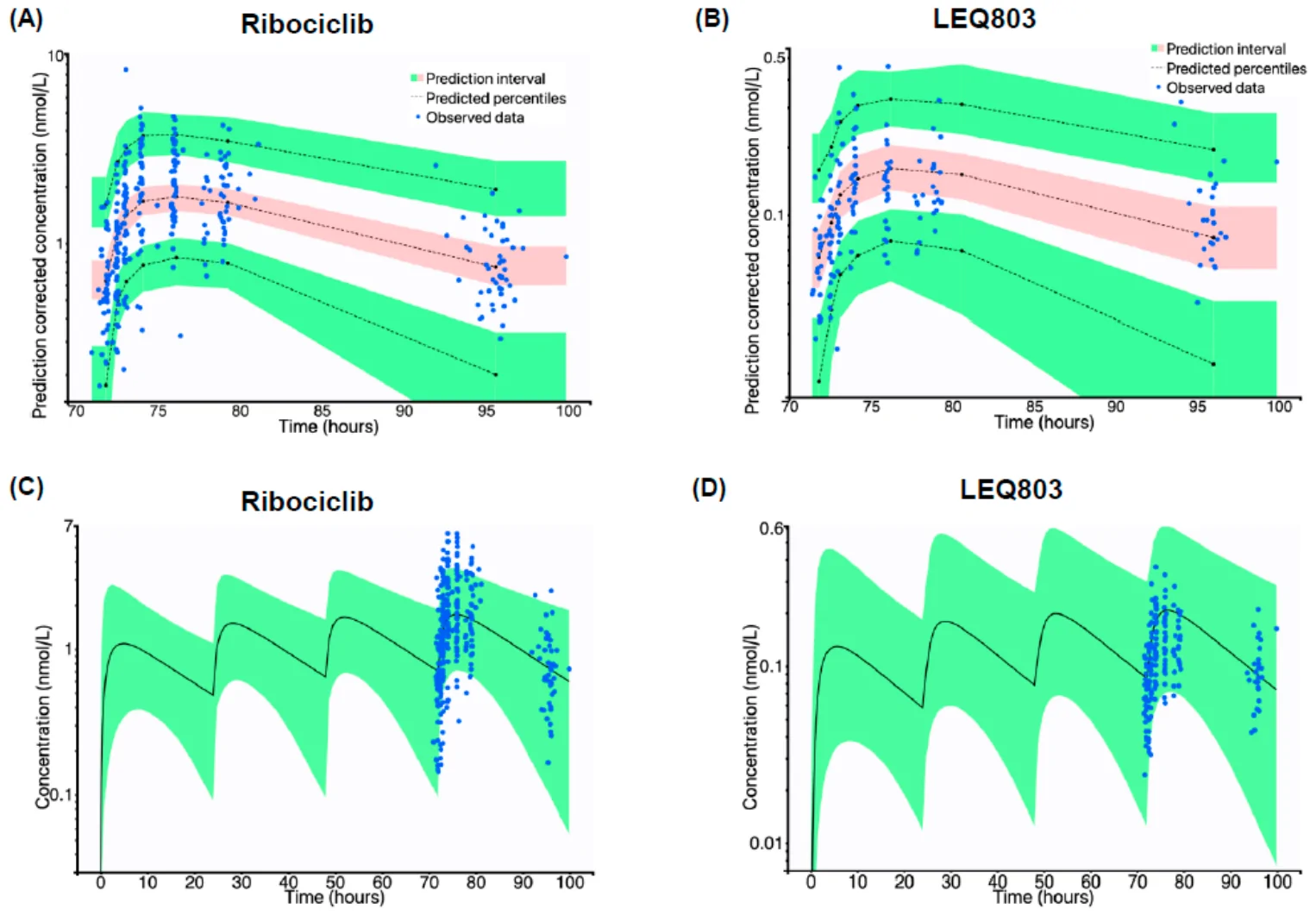
Population pharmacokinetic model evaluation. (**A**,**B**) Visual predictive check from the final covariate model for ribociclib and LEQ803. The dashed lines represent the 50th, 5th, and 95th percentiles of the model-predicted concentration–time profiles. The shaded areas represent the 95% confidence intervals of the percentiles. Symbols represent the observed drug concentration data. (**C**,**D**) Prediction distribution plots for ribociclib and LEQ803 from the final covariate model. The solid lines represent the model predicted population mean drug concentration–time profile, and the shaded areas represent the 95% confidence intervals. Dot symbols represent observed concentration data.

The predictive performance of the final covariate model was rigorously evaluated by goodness-of-fit plots, nonparametric bootstrap analysis, visual predictive checks (VPCs), and prediction distribution plots (Figure 3 and Figure 4). Goodness-of-fit plots (Figure 3) indicated strong agreement between observed and model-predicted plasma concentrations of ribociclib and LEQ803 and randomly distributed residuals around the horizontal zero line, suggesting the absence of systematic prediction bias. The VPC plots (Figure 4A,B) suggested that the observed plasma concentrations of ribociclib and LEQ803 were largely contained within the 95% prediction intervals and were in good agreement with the simulated median (50th percentile) and variability bounds (5th and 95th percentiles), indicating that the model adequately captured both the central tendency and variability of the observed data. Prediction distribution plots (Figure 4C,D) demonstrated that the observed data were well contained within the 95% prediction intervals and closely aligned with the simulated percentiles, further supporting the absence of model misspecification.

Table 2 summarizes population pharmacokinetic parameters of ribociclib and LEQ803 along with their respective interindividual variabilities, as estimated from the base model and final covariate model. The robustness of the parameter estimates was further supported by bootstrap analysis. As summarized in Table 3, the population mean pharmacokinetic parameter estimates obtained from the final covariate model were consistent with the mean estimates derived from the bootstrap replicates, and both fell within the 95% confidence intervals of the bootstrap estimates, confirming the stability and reliability of parameter estimation.

Following oral administration, ribociclib was absorbed into the systemic circulation with a population mean first-order absorption rate constant (Ka) of 0.52 h^−1^ and exhibited extensive distribution, with a population mean apparent volume of distribution (Vp/F) of ~996 L (Table 2). Ribociclib was predominantly metabolized by hepatic CYP3A4 to form its major metabolite, LEQ803, with a population mean metabolic clearance (CLpm) of 6.48 L/h. The total apparent oral clearance of ribociclib (CLp/F), calculated as the sum of CLpm and CLp/F_other_, was estimated to be 58.4 L/h (Table 2), which was consistent with the mean apparent oral clearance (57.5 L/h) estimated from non-compartmental analysis. Compared with the parent drug, LEQ803 exhibited a more limited distribution, with a population mean volume of distribution of 36.3 L, while maintaining a comparable systemic clearance (population mean, 53.1 L/h) (Table 2). Notably, the model-estimated population mean elimination rate constant and formation rate constant of LEQ803 were 1.463 h^−1^ and 0.0065 h^−1^, respectively, suggesting that systemic exposure to LEQ803 was formation-rate limited by the metabolic conversion of ribociclib.

### 3.3. Clinical Covariates Influencing Ribociclib Metabolism and Plasma Pharmacokinetics

Generalized additive model (GAM) analysis followed by a stepwise covariate model-building process demonstrated that patient demographics (race, sex, age, and brain tumor type), body size measures (body weight and body surface area), plasma albumin levels, hepatic function markers (ALT, AST, and total bilirubin), and renal function markers (serum creatinine and creatinine clearance) were not statistically significant covariates on any pharmacokinetic parameters of either ribociclib or LEQ803. These findings were consistent with previously published population pharmacokinetic analysis of ribociclib [31].

In vivo hepatic CYP3A activity, assessed by plasma 4β-OHC concentrations following ribociclib treatment, was identified as a significant covariate on the metabolic clearance (CLpm) governing the conversion of ribociclib to LEQ803. Specifically, incorporation of the 24 h plasma 4β-OHC concentration as an exponential function on CLpm resulted in a decrease in OFV of 12.54 (*p* < 0.001) and reduced the interindividual variability of CLpm from 34% in the base model to 22% in the final covariate model, indicating that the 24 h plasma 4β-OHC concentration explained approximately 36% of the interindividual variability in CLpm (Table 2). Interestingly, patient height was identified as a statistically significant covariate on the apparent clearance via other pathways (CLp/F_other_), but it only explained ~5.3% of the interindividual variability in CLp/F_other_, suggesting patient height was unlikely to be a clinically significant covariate.

**Figure 5:**
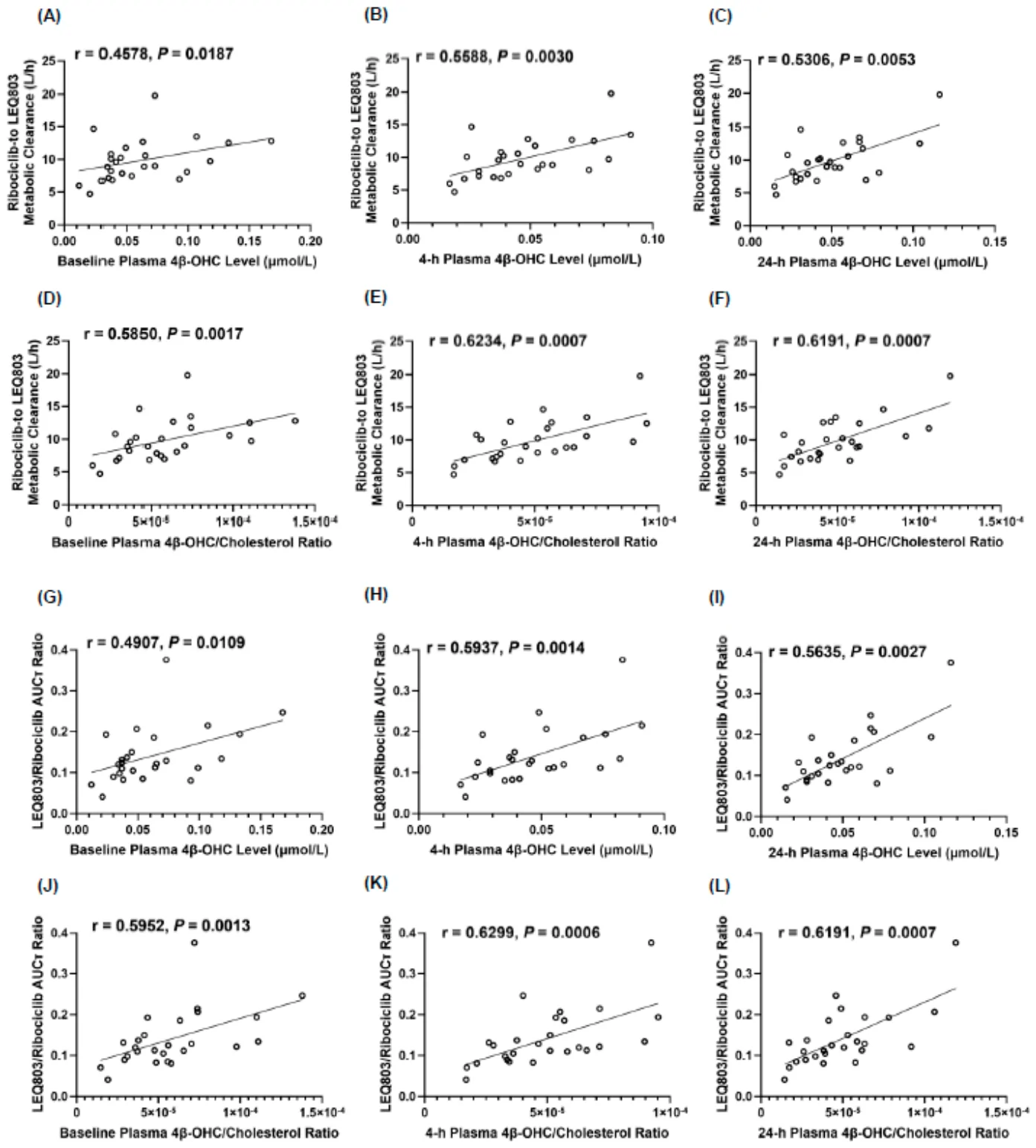
Spearman’s rank correlation analysis of the association between endogenous biomarkers of hepatic CYP3A4 activity and CYP3A-mediated ribociclib metabolism, assessed either by the population pharmacokinetic base model–estimated metabolic clearance (CLpm) (**A**–**F**) or by the trapezoidal method–estimated LEQ803-to-ribociclib AUCτ ratio (**G**–**L**).

**Figure 6:**
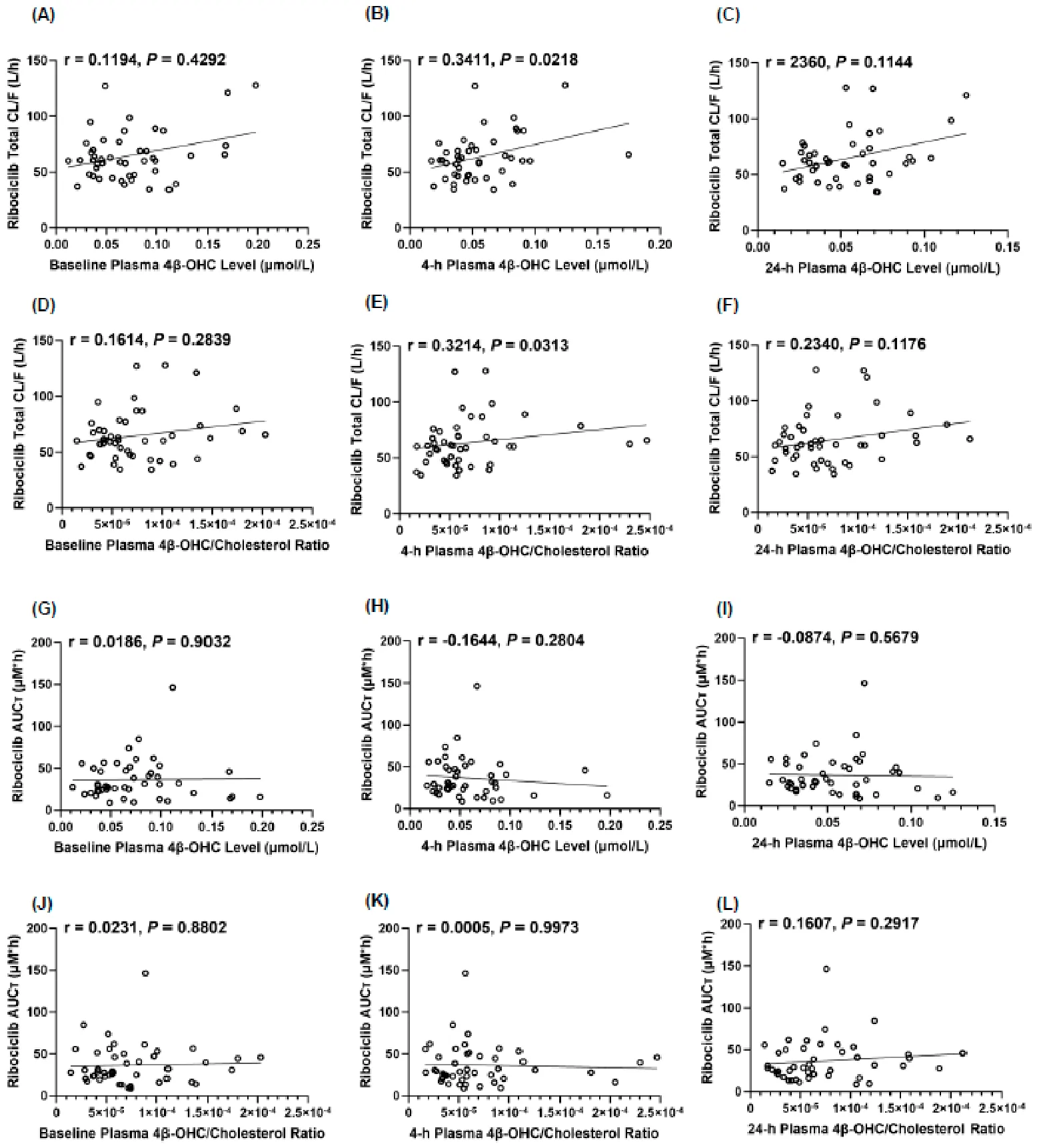
Spearman’s rank correlation analysis of the association between endogenous biomarkers of hepatic CYP3A4 activity and ribociclib systemic pharmacokinetics, assessed either by the population pharmacokinetic base model–estimated total apparent oral clearance (**A**–**F**) or by the trapezoidal method–estimated ribociclib AUCτ (systemic exposure) (**G**–**L**).

The association between ribociclib metabolic conversion to LEQ803 and endogenous biomarkers of hepatic CYP3A4 activity was further evaluated using Spearman’s rank correlation analysis (Figure 5). CYP3A-mediated ribociclib metabolism, assessed either by the population pharmacokinetic base model–estimated metabolic clearance (CLpm) (Figure 5A–F) or by the trapezoidal method–estimated LEQ803-to-ribociclib AUCτ ratio (Figure 5G–L), showed statistically significant correlations with all evaluated markers of hepatic CYP3A4 activity, with stronger correlations observed for on-treatment plasma 4β-OHC concentrations and 4β-OHC/cholesterol ratios than for the corresponding baseline measurements. In contrast, total apparent oral clearance of ribociclib (CL/F), calculated as the sum of CLpm and CLp/F_other_ from the population pharmacokinetic base model, showed either no correlation or only weak statistically significant correlations with hepatic CYP3A4 activity markers (Figure 6A–F); and consistently, trapezoidal method–estimated ribociclib AUCτ was not significantly correlated with any of the evaluated hepatic CYP3A4 activity markers (Figure 6G–L). Collectively, the population pharmacokinetic and Spearman’s rank correlation analyses demonstrated that on-treatment plasma 4β-OHC concentration, as an endogenous marker of hepatic CYP3A activity, was a significant predictor of CYP3A-mediated ribociclib metabolism, while it has limited utility for predicting the total apparent oral clearance or systemic plasma exposure of ribociclib.

## 4. Discussion

In this study, a parent–metabolite population pharmacokinetic model was developed that adequately characterized the plasma concentration–time profiles of ribociclib and its major metabolite, LEQ803, in patients with primary brain cancer. The model enabled separation of the metabolic clearance responsible for ribociclib conversion to LEQ803 from other elimination pathways, thereby allowing assessment of covariate effects on the specific CYP3A-mediated metabolic pathway, other clearance pathways, and the total apparent oral clearance of ribociclib. Among all evaluated clinical covariates (Table 1 and Figure 2), the endogenous biomarker of in vivo hepatic CYP3A activity, particularly the plasma 4β-OHC concentration measured 24 h after ribociclib administration, was identified as a statistically significant and clinically relevant covariate on the metabolic clearance (CLpm) governing ribociclib conversion to LEQ803. Inclusion of this covariate explained approximately 36% of the interindividual variability in CLpm (Table 2). The significant correlation between ribociclib metabolic conversion to LEQ803 and endogenous biomarkers of hepatic CYP3A4 activity was further confirmed by Spearman’s rank correlation analysis (Figure 5). Collectively, these findings support the potential translational utility of plasma 4β-OHC concentration as an endogenous biomarker of hepatic CYP3A activity for predicting CYP3A-mediated ribociclib metabolism.

Notably, ribociclib is a time-dependent inhibitor of CYP3A [10], as evidenced by lower plasma 4β-OHC concentrations or 4β-OHC/cholesterol ratios after ribociclib administration on Day 5 compared with pretreatment baseline values (Figure 2). Both the population pharmacokinetic analysis and Spearman’s rank correlation analysis consistently identified the on-treatment plasma 4β-OHC concentration as the stronger predictor of CYP3A-mediated ribociclib metabolism. These findings underscore the importance of longitudinal monitoring of hepatic CYP3A activity during ribociclib treatment and suggest that on-treatment plasma 4β-OHC concentration provides a more reliable predictor of CYP3A-mediated ribociclib metabolism than baseline measurement.

In contrast, neither the population pharmacokinetic analysis nor the Spearman correlation analysis identified any evaluated measure of in vivo hepatic CYP3A activity as a statistically significant predictor for the total apparent oral clearance or systemic plasma exposure of ribociclib (Figure 6). These findings are not unexpected, as pathways independent of hepatic CYP3A activity also contribute substantially to the overall disposition of ribociclib [9]. The parent–metabolite population pharmacokinetic model predicted that the metabolic clearance associated with ribociclib conversion to LEQ803 accounts for approximately 11% of the total apparent oral clearance of ribociclib, calculated as 6.48/(6.48 + 51.90), whereas all other elimination pathways collectively account for the remaining ~89% (Table 2). This prediction is in line with published in vitro and in vivo metabolism and pharmacokinetic data of ribociclib [9]. A mass balance study in humans demonstrated that ~69.1% and 22.6% of the radiolabeled dose were recovered in feces and urine, respectively; and of the administered dose, 17.3% and 6.75% were excreted as unchanged drug in feces and urine, respectively, with the remainder recovered as multiple metabolites [9]. In vitro and in vivo metabolism studies have further shown that ribociclib undergoes extensive hepatic metabolism, predominantly mediated by CYP3A4 to form LEQ803 and other oxidative metabolites; and in addition, it is metabolized, to a lesser extent, by flavin-containing monooxygenase 3 (FMO3) via *N*-hydroxylation, while additional hepatic enzymes. including CYP1A2, CYP2J2, and CYP3A5, make a minor contribution [9]. Taken together, ribociclib elimination occurs primarily through hepatic metabolism, accounting for approximately 75% of total clearance, with additional contributions from renal excretion (7%), intestinal excretion (17%), and biliary elimination (1%) [9]. Therefore, the absence of a significant association between in vivo hepatic CYP3A activity biomarkers and ribociclib total apparent oral clearance is likely attributable to the substantial contribution of non-CYP3A-mediated elimination pathways. Although CYP3A4 plays a dominant role in the formation of LEQ803, multiple metabolic enzymes, together with renal, intestinal, and biliary elimination processes, contribute to the overall disposition of ribociclib, thereby diminishing the influence of hepatic CYP3A activity on total drug exposure.

In addition, it is important to recognize that 4β-OHC is produced predominantly in the liver, limiting its ability to reflect total CYP3A activity across tissues, particularly in the intestine [20]. Ribociclib is moderately to highly absorbed across species, with an estimated oral bioavailability of approximately 59% in humans [9,31]. Consequently, a substantial fraction of the orally administered dose is likely exposed to intestinal first-pass metabolism, primarily mediated by CYP3A enzymes, before reaching the systemic circulation. This hypothesis is supported by preclinical studies in rats and dogs, which demonstrated a higher LEQ803-to-ribociclib AUC ratio following oral administration than after intravenous administration, indicating intestinal first-pass metabolism [9]. Because 4β-OHC is generated almost exclusively in the liver and does not adequately capture intestinal CYP3A activity, its utility as a biomarker for predicting intestinal first-pass metabolism is inherently limited [20]. Therefore, the inability of plasma 4β-OHC concentrations to reflect intestinal CYP3A activity likely further contributes to its poor performance in predicting oral bioavailability, total apparent oral clearance, and systemic exposure of ribociclib.

## 5. Conclusions

In conclusion, the parent–metabolite population pharmacokinetic model adequately characterized the multiple-dose plasma concentration–time profiles of ribociclib and its major metabolite, LEQ803, in patients with primary brain cancer. The model demonstrated that plasma 4β-hydroxycholesterol (4β-OHC), an endogenous biomarker of hepatic CYP3A activity, is a significant predictor of CYP3A-mediated ribociclib metabolism to LEQ803. In contrast, plasma 4β-OHC showed limited ability to predict the total apparent oral clearance or systemic exposure of ribociclib. These findings support the clinical utility of plasma 4β-OHC as an endogenous biomarker of hepatic CYP3A activity for predicting CYP3A-mediated hepatic metabolism of substrate drugs such as ribociclib. However, plasma 4β-OHC alone appears insufficient to predict total apparent oral clearance or systemic drug exposure for compounds that undergo intestinal first-pass metabolism and are eliminated through multiple pathways in addition to hepatic CYP3A-mediated metabolism. More broadly, these results highlight the importance of considering the relative contribution of hepatic CYP3A metabolism to overall drug disposition when evaluating the translational utility of endogenous hepatic CYP3A biomarkers in clinical pharmacology.

## Figures and Tables

**Table 1:** Patient demographic and characteristic data.

	Median (Range) or Number of Patients
Baseline characteristics	
Race (white/non-white) *	44/2
Sex (male/female) *	30/16
Age (years)	58 (27–72)
Weight (kg)	82 (54–115)
Height (cm)	175 (155–198)
BSA (m^2^)	1.99 (1.52–2.49)
Liver function	
Predose total bilirubin (mg/dL)	0.7 (0.3–1.9)
Post-operation total bilirubin (mg/dL)	0.5 (0.3–1.4)
Predose AST (IU/L)	20 (11–115)
Post-operation AST (IU/L)	20 (12–106)
Predose ALT (IU/L)	23 (7–231)
Post-operation ALT (IU/L)	30 (7–419)
Predose plasma albumin (mg/dL)	4.4 (3–5)
Post-operation plasma albumin (mg/dL)	3.8 (2.5–4.6)
Kidney function	
Predose serum creatine (mg/dL)	0.865 (0.51–1.49)
Post-operation serum creatine (mg/dL)	0.8 (0.52–1.69)
Predose creatine clearance (mL/min)	105.93 (68.74–269.65)
Post-operation creatine clearance (mL/min)	113.58 (63.46–264.46)
Concomitant drugs	
Co-administered drugs during the trial (yes/no) *	46/0

* Data indicates number of patients. Abbreviations: AST, aspartate aminotransferase; ALT, alanine aminotransferase.

**Table 2:** Population pharmacokinetic parameters estimated from the base (structure) model and final covariate model.

Parameter *	Base Model	Final Model
OFV	−2548.15	−2567.16
TV Ka ($h^{-1}$)	0.51	0.52
TV Vp/F (L)	982.74	995.61
TV Vm (L)	36.34	23.39
TV CLp/F_other_ (L/h)	50.8	51.90 ^†^
TV CLpm (L/h)	9.31	6.48 ^‡^
TV CLm (L/h)	79.75	53.14
$\theta_{1}\;(Ka)$ (R.S.E)	0.51 (27.6)	0.52 (19.7)
$\theta_{2}\;(Vp/F)$ (R.S.E)	982.74 (17.4)	995.61 (11.0)
$\theta_{3}\;(Vm)$ (R.S.E)	36.34 (46.9)	23.39 (20.2)
$\theta_{4}\;(CLp/Fother)$ (R.S.E)	50.8 (6.36)	51.38 (5.85)
$\beta_{1}$ (Height on CLp/Fother)	-	2.04 (43.7)
$\theta_{5}\;(CLpm)$ (R.S.E)	9.31 (18.5)	3.57 (17.6)
$\beta_{2}\;(4\beta OHC$_24 h on Clpm)	-	11.35 (27.7)
$\theta_{6}\;(CLm)$ (R.S.E)	79.75 (18.3)	53.14 (15.1)
IIV of Ka % (R.S.E)	100 (41.6)	112 (26.1)
IIV of Vp/F % (R.S.E)	46 (65.6)	37 (33.2)
IIV of Vm % (R.S.E)	32 (79.3)	26 (74.0)
IIV of CLp/Fother % (R.S.E)	40 (13.0)	38 (12.9)
IIV of CLpm % (R.S.E)	34 (23.9)	22 (36.1)
IIV of CLm % (R.S.E)	18 (39.7)	20 (49.4)

* PK parameter estimates are expressed as the population mean (% relative standard error of estimation). ^†^ TV_CLp/F_other_ = θ_4_ × (Height/median)^β1^, with the population median height being 175.26. ^‡^ TV_CLpm = θ_5_ × e^β2^ × ^4βOHC_24h^, with the population median 4βOHC 24 h being 0.05248. Abbreviations: θ, population mean PK parameter estimate from the final covariate model; TV, typical population mean PK parameter; Ka, absorption rate constant; CLp/F_other_, apparent clearance via other pathways; Vp/F, apparent volume of distribution for parent drug; CLm, systemic clearance for metabolite; Vm, volume of distribution for metabolite; CLpm, metabolic clearance for ribociclib conversion to LEQ803; IIV, interindividual variability.

**Table 3:** Population pharmacokinetic parameters estimated from the final covariate model and bootstrap analysis.

Parameter	Population Mean	Bootstrap Mean	Bootstrap 95% CI
$\theta_{1}$ (Ka) (h^−1^)	0.52	0.52	(0.33, 0.71)
$\theta_{2}$ (Vp/F) (L)	995.61	1021.42	(766.28, 1321.39)
$\theta_{3}$ (Vm) (L)	23.39	22.47	(15.21, 32.67)
$\theta_{4}$ (CLp/Fother) (L/h)	51.38	50.46	(44.81, 57.06)
$\theta_{5}$ (CLm) (L/h)	53.14	54.02	(41.31, 69.12)
$\theta_{6}$ (CLpm) (L/h)	3.57	3.87	(2.53, 6.01)
$\beta_{1}$ (Height)	2.04	2.25	(0.32, 4.4)
$\beta_{2}$ (OH_cholesterol_24h)	11.35	10.23	(2.91, 15.99)
Ka_IIV	0.9	0.82	(0.37, 1.45)
Vp/F_IIV	0.36	0.42	(0.21, 0.69)
Vm_IIV	0.26	0.24	(0.12, 0.44)
CLp/Fother_IIV	0.37	0.33	(0.24, 0.41)
CLm_IIV	0.2	0.19	(0.084, 0.34)
CLpm_IIV	0.24	0.19	(0.076, 0.32)

Abbreviations: θ, population mean PK parameter estimate from the final covariate model; Ka, absorption rate constant; CLp/F_other_, apparent clearance via other pathways; Vp/F, apparent volume of distribution for parent drug; CLm, systemic clearance for metabolite; Vm, volume of distribution for metabolite; CLpm, metabolic clearance for ribociclib conversion to LEQ803; IIV, interindividual variability.

## Data Availability

The data generated in this study are available within the paper. The raw data used to create graphs are available from the corresponding author upon reasonable request.
